# Primary ovarian insufficiency consequence of autoimmune diseases: a bidirectional two-sample Mendelian randomization study

**DOI:** 10.3389/fendo.2024.1417896

**Published:** 2024-12-09

**Authors:** Yongming Du, Yichao Hu, Yuehua Sheng, Tianhong Zhu, Shenping Liu, Huiqing Ding, Yutao Guan

**Affiliations:** ^1^ Department of Obstetrics and Gynecology, The First Affiliated Hospital of Ningbo University, Ningbo, China; ^2^ Department of Urology, The First Affiliated Hospital of Ningbo University, Ningbo, China

**Keywords:** autoimmune disease, primary ovarian insufficiency, Mendelian randomization, single-nucleotide polymorphisms, causal association

## Abstract

**Background:**

Observational studies suggest the risk of primary ovarian insufficiency (POI) is increased in autoimmune disorders (AIDs), but it is unclear whether there is a causal relationship. Therefore, we aimed to investigate the bidirectional causality between 20 AIDs and POI using Mendelian randomization (MR) analysis.

**Methods:**

A bidirectional two-sample MR investigation was designed by using publicly accessible summary-level data from genome-wide association studies (GWAS). The inverse variance weighted (IVW) method was performed as the main analysis, supplemented by several sensitivity analyses. Cochran Q test was used to evaluate SNP estimate heterogeneity. MR-Egger and MR-PRESSO methods were utilized to detect horizontal pleiotropy.

**Results:**

The MR analyses revealed that genetically determined coeliac disease (CeD) (OR = 1.124, 95% CI 1.033-1.224, P = 0.007), vitiligo (OR = 1.092, 95% CI 1.003-1.188; P = 0.042), systemic lupus erythematosus (SLE) (OR = 1.122, 95% CI 1.030-1.223, P = 0.008), and selective immunoglobulin A deficiency (SIgAD) (OR = 0.866, 95% CI: 0.776-0.967, P = 0.011) exhibited significant causal relationships with POI. We also found suggestive evidence of positive effect of Addison’s disease (AD) towards POI (OR_5e-6_ = 1.076, 95% CI 1.002-1.154, P = 0.043).

**Conclusion:**

This comprehensive MR analysis indicated that SLE, CeD, vitiligo, and AD caused an increased risk of POI, SIgAD was associated with a decreased risk of POI. These insights carry profound clinical implications, particularly emphasizing the early intervention for women with AIDs/POI who wish to preserve their reproductive potential or plan for future pregnancies.

## Introduction

Primary ovarian insufficiency (POI) is a disorder characterized by the premature cessation of ovarian function before the age of 40, leading to amenorrhea, low estrogen levels, and elevated follicle-stimulating hormone (FSH) levels (1). The incidence of POI within the general female demographic is approximated to range from 1% to 3.7% (2–5). The state of early estrogen deficiency caused women facing an elevated risk for a spectrum of health complications, including cardiovascular disease, dementia, declined neurocognitive function, osteoporosis, and augmented overall mortality rate (6–11). POI arises from a complex interplay of genetic and immunological determinants, yet a substantial proportion of instances are idiopathic (12). The contribution of autoimmunity to POI is variably estimated between 5-30%, highlighting the cohort heterogeneity in the studied populations (12–15).

Autoimmune diseases (AIDs), such as thyroid disorders (Hashimoto thyroiditis and Grave’s disease) (16), Addison’s disease (17), and systemic lupus erythematosus (18), have been implicated in the pathogenesis of POI. However, the complex interplay between these conditions and POI remains incompletely understood, and the causal relationships are not fully elucidated. Traditional observational studies have been limited by potential confounders and reverse causation, which complicate the determination of causality in the relationship between autoimmune diseases and POI. Additionally, for patients presenting with POI in gynecological endocrinology clinics, the European Society of Human Reproduction and Embryology (ESHRE) has issued guidelines in screening for 21OH-Ab/ACA or TPO-Ab/TSH tests to identify certain immune disorders (1). However, in the case of women of reproductive age attending rheumatology departments due to autoimmune diseases, there currently exist no consensus or guidelines specifically dedicated to the screening of POI within this patient population. Early detection and diagnosis of POI is crucial for timely intervention, preservation of fertility, and management of long-term health risks. Indiscriminate surveillance and monitoring are anticipated to contribute to an augmented workload in the management of POI.

The current study employs Mendelian randomization (MR), a powerful epidemiological tool, to overcome the limitations of traditional observational research and provide novel insights into the causal interplay between a spectrum of autoimmune disorders and POI (19–21). By utilizing genetic variants as instrumental variables, MR allows for the circumvention of confounding factors and the establishment of causal inferences. This robust design ensures that the observed associations are less likely to be influenced by lifestyle factors, environmental exposures, or reverse causation, thereby offering a more accurate representation of the relationship between autoimmune disorders and the risk of POI. To our knowledge, comprehensive overview of the primary ovarian insufficiency consequence of autoimmune diseases is currently lacking. In this context, we have conducted a two-sample bidirectional MR study to systematically investigate the causal association between autoimmune diseases and POI. The significance of this research lies in its potential to inform clinical practice, particularly in the early identification and intervention for women with autoimmune diseases who wish to preserve their reproductive potential or are planning for future pregnancies.

## Methods

### Study design

**Figure 1:**
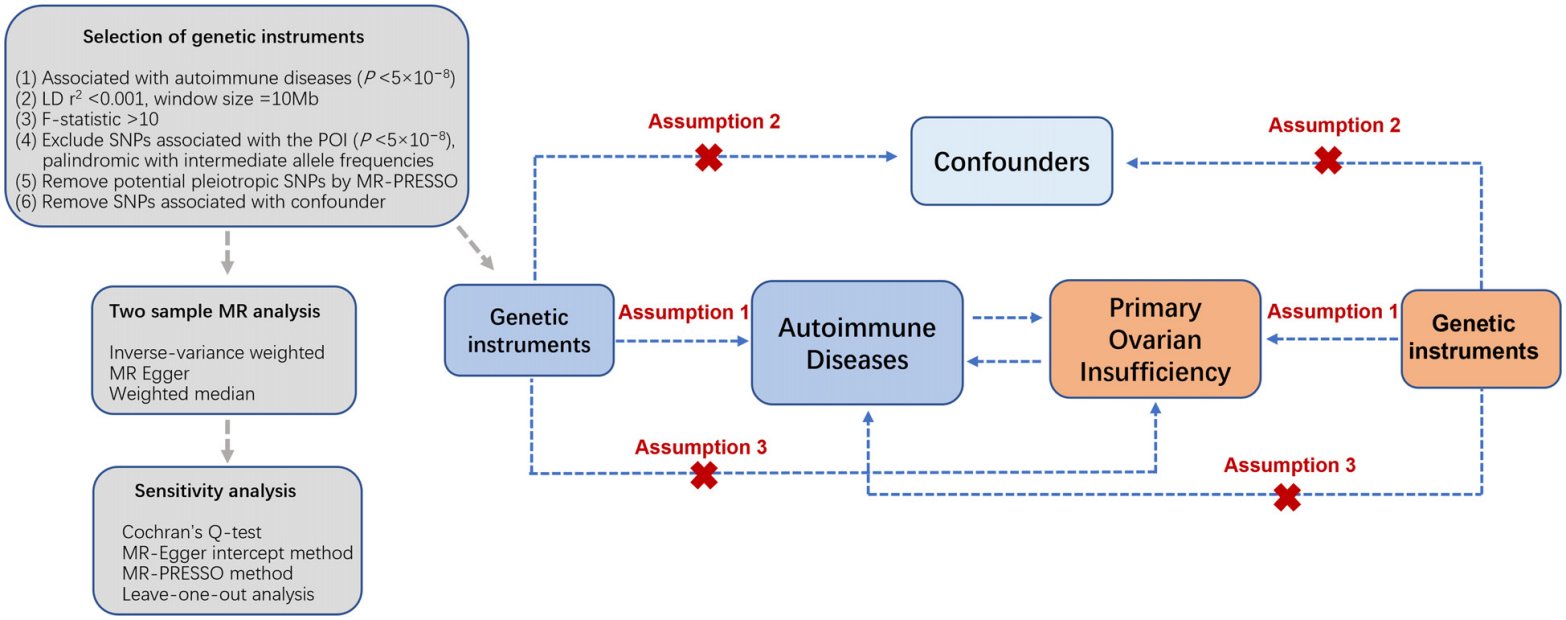
Overview of the bidirectional two-sample MR analysis. Assumption 1: genetic instrumental variables are strongly related to the exposure; Assumption 2: genetic instrumental variables are independent from confounding factors of the exposure-outcome; Assumption 3: genetic instrumental variables influence outcomes solely through the exposure. SNPs, single nucleotide polymorphisms; POI, primary ovarian insufficiency; LD, linkage disequilibrium.

The bidirectional two-sample MR investigation was designed to elucidate the causal association of 20 autoimmune diseases on the susceptibility to POI (**Figure 1**) following the Strengthening the Reporting of Observational Studies in Epidemiology Using Mendelian Randomization (STROBE-MR) guidelines (19). The investigation relied upon publicly accessible summary-level data derived from genome-wide association studies (GWAS), specifically incorporating datasets from the websites of FinnGen study (https://www.finngen.fi/en/access_results), GWAS Catalog (https://www.ebi.ac.uk/gwas/) (22), and other expansive consortia, ensuring the absence of overlap in study populations. Subsequently, we identified and utilized an additional contemporary dataset on autoimmune diseases from the IEU GWAS repository (https://gwas.mrcieu.ac.uk/datasets/) to corroborate our analytical findings. All incorporated studies underwent ethical scrutiny and received approval from pertinent review boards, with participants providing informed consent.

### Instrumental variables

#### Genetic variants associated with autoimmune diseases

Our investigation primarily aimed to explore the causal associations between 20 of the most common autoimmune disorders and POI. Autoimmune diseases included type 1 diabetes mellitus (T1DM) (23), coeliac disease (CeD) (24), Crohn’s disease (CD) (25), primary biliary cholangitis (PBC) (26), ulcerative colitis (UC) (27), Hashimoto’s thyroiditis (HT) (27), Grave’s disease (GD) (27), hypothyroidism (28), Addison’s disease (AD) (29), polymyalgia rheumatica (PMR) (30), myasthenia gravis (MG) (31), rheumatoid arthritis (RA) (27), multiple sclerosis (MS) (32), Sjogren’s syndrome (SS) (33, 34), psoriasis (35), vitiligo (36), systemic sclerosis (SSc) (37), idiopathic thrombocytopenic purpura (ITP) (27), systemic lupus erythematosus (SLE) (38), and selective immunoglobulin A deficiency (SIgAD) (39). To obtain the comprehensive information of the exposures among the European population, we identified and selected the most extensive and contemporaneously updated GWAS meta-analysis study for the initial MR analysis, as delineated in **Table 1**.

**Table 1:** Detailed information for the GWAS data.

Exposures	GWAS ID	Data Source	Sample Size	Cases/Controls	Year	Ancestry
Type 1 diabetes mellitus	GCST010681	Forgetta V et al.	520,580	18,942/501,638	2021	European
Coeliac disease	GCST005523	Trynka et al.	24,269	12,041/12,228	2011	European
Crohn's disease	GCST004132	de Lange et al.	40,266	12,194/28,072	2017	European
Primary biliary cholangitis	GCST003129	Cordell et al.	13,239	2,764/10,475	2015	European
Ulcerative colitis	GCST90018933	Sakaue et al.	417,932	5,371/412,561	2021	European
Hashimoto's thyroiditis	GCST90018855	Sakaue et al.	395,640	15,654/379,986	2021	European
Grave's disease	GCST90018847	Sakaue et al.	458,620	1,678/456,942	2021	European
Hypothyroidism	GCST90204167	Mathieu et al.	494,577	51,194/443,383	2022	European
Addison's disease	GCST90011871	Eriksson et al.	5,320	1,223/4,097	2021	European
Polymyalgia rheumatica	GCST90129454	Zorina-Lichtenwalter et al.	435,971	2,460/433,511	2023	European
Myasthenia gravis	GCST90093061	Chia et al.	38,243	1,873/36,370	2022	European
Rheumatoid arthritis	GCST90018910	Sakaue et al.	417,256	8,255/409,001	2021	European
Multiple sclerosis	ieu-b-18	International Multiple Sclerosis Genetics Consortium	115,803	47,429/68,374	2019	European
Sjogren's syndrome	GCST90018920	Sakaue et al.	484,013	1,296/482,717	2017	European
Psoriasis	GCST004346	Tsoi L et al.	34,772	13,229/21,543	2017	European
Vitiligo	GCST007111 GCST007112	Jin et al.	30,358	2,171/28,187	2019	European
Systemic sclerosis	GCST90319682	López-Isac et al.	26,679	9,095/17,584	2024	European
Idiopathic thrombocytopenic purpura	GCST90018865	Sakaue et al.	489,424	675/488,749	2021	European
Systemic lupus erythematosus	GCST003156	Bentham et al.	23,210	7,219/15,991	2015	European
Selective immunoglobulin A deficiency	GCST003814	Bronson et al.	6,487	1,635/4,852	2016	European
Outcome	GWAS ID	Data Source	Sample Size	Cases/Controls	Year	Ancestry
Primary ovarian insufficiency	finn-b-E4_OVARFAIL	FinnGen	219,512	542/218,970	2023	Finnland

#### Genetic variants associated with POI

The GWAS data for primary ovarian insufficiency was obtained from the European FinnGen project, a study identifying genotype-phenotype correlations in 500,000 Finnish biobank participants (40). We used the recently updated R10 data release of FinnGen, encompassing 542 cases of POI and 218,970 controls. The inclusion criteria of POI came from the codes of the International classification of diseases 10th Revision (ICD-10), ICD-9, and ICD-8 with a specific phenotypic code being “E4_OVARFAIL”. The European Society of Human Reproduction and Embryology (ESHRE) criteria are employed for the diagnosis of POI. These criteria comprise the individual under 40 years, getting rare or absent menstruation for at least four months, and with at least two serum basic follicle-stimulating hormone (FSH) tests with levels exceeding 25 U/L, executed at least four weeks out (1).

### Instrumental variables selection

Single-nucleotide polymorphisms (SNPs) associated with autoimmune disorders, reaching genome-wide significance (P < 5×10^−8^), were meticulously curated from the extensive GWAS studies. Linkage disequilibrium (LD) among SNPs associated with each autoimmune disease was computed utilizing the 1000 Genomes European panel and the clumping method. SNPs exhibiting linkage disequilibrium (defined as r^2^ > 0.001 and clump distance <10,000 kb) were excluded from the analysis (41). The strength of the instrumental variables was evaluated through the utilization of F-statistic. $F=\left(\frac{n-k-1}{k}\right)\left(\frac{R^{2}}{1-R^{2}}\right)$ , wherein R^2^ denotes the proportion of variability that can be explained by IVs in the exposure, n signifies the sample size, and k represents the number of SNPs (42). IVs were designated as weak if the F-statistic was below 10 and were consequently omitted (43). We also excluded IVs that had a pronounced correlation with the POI (P < 5×10^−8^). Additionally, IVs that were palindromic with intermediate allele frequencies were disregarded. SNPs flagged as outliers by MR-PRESSO were similarly excluded to maintain the robustness of the MR analysis (44). Cigarette smoking, a common environmental determinant and established risk factor for POI development (45), had its associated SNPs removed (46). In the forward and reverse MR analyses for genetic variation selection, the standard practice is to incorporate all genome-wide significant variants (P < 5×10^−8^) to minimize false positives. In the absence of such variants or in the cases with limited number of SNPs, alternative thresholds (e.g., 5×10^−6^, 1×10^−5^) was employed, provided they adhere to the instrumental variable criteria.

### Statistical analysis

Two-sample MR analysis was executed bidirectionally, initially considering the associations between genetic predisposition to 20 common autoimmune disorders with the risk of POI. The second set of MR analyses handled POI as the exposure and autoimmune disorders as the outcomes. A comparative assessment across three distinct methods was performed, encompassing inverse-variance weighted (IVW) (47), MR Egger (48), and weighted median (47). Each method incorporates slightly different assumptions regarding the nature of pleiotropy, thus a consistent point estimate across multiple methods enhances the robustness of causal inference, with the IVW method serving as primary analysis and other two methods contributing as sensitivity analyses. Cochran Q-test was utilized to evaluate heterogeneity across MR analysis (49). In instances where considerable heterogeneity was observed, we employed the IVW random-effects model to address this variability. We conducted a suite of sensitivity analyses encompassing the weighted median, MR-Egger, and MR-PRESSO methods to ascertain the stability of our findings and to detect potential horizontal pleiotropy. The weighted median estimation in MR analysis is reliable when a majority of the contributing weight is attributed to SNPs without pleiotropic influences. The MR-Egger regression, equipped with an intercept test, discerns and rectifies horizontal pleiotropy, yielding adjusted effect estimates. The MR-PRESSO approach identifies and rectifies pleiotropic outliers and employs a global test to assess SNP heterogeneity indicative of pleiotropy. A p-value threshold of > 0.05 was established to denote a lack of heterogeneity or pleiotropy among the SNPs. Additionally, we conducted a leave-one-out analysis to scrutinize the influence of outlier and pleiotropic SNPs on the causal estimates (50). Analyses of the POI against autoimmune diseases used a Bonferroni multiple testing corrected P-value of 0.0025 (0.05/20) because twenty autoimmune disorders were examined. Threshold values ranging from 0.0025 to 0.05 were interpreted as suggestive of potential causal relationships while adjusted P values < 0.0025 were deemed significant. All analyses were conducted using R packages TwoSampleMR and MRPRESSO in R version 4.1.3.

## Results

### Genetic instruments and strength

**Figure 2:**
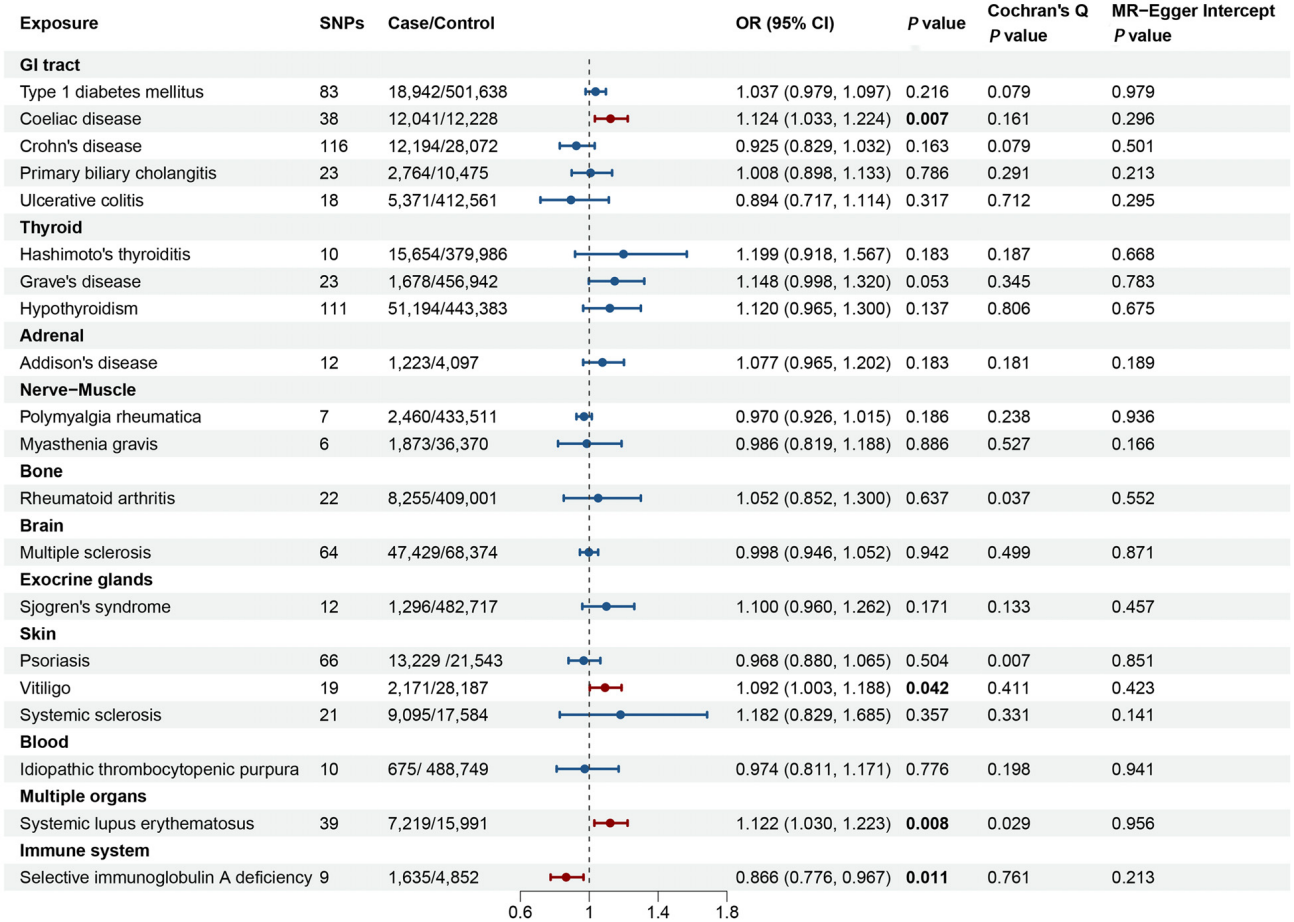
Forest plot illustrating the causal effect of autoimmune diseases on primary ovarian insufficiency. The causal effect of different autoimmune diseases on primary ovarian insufficiency was expressed as OR per unit. Error bars represent the 95% CIs of the estimates. GI, gastrointestinal; SNPs, single nucleotide polymorphisms; OR, odds ratio; CI, confidence interval.

Each single SNP exhibited an F-statistic value that surpassed the critical threshold of 10, thereby mitigating the potential confounding effects of weak instrumental variables on the analytical outcomes. Detailed information of the individual SNP characteristics is provided in **Supplementary Tables 1**–**20**. The synthesized MR analyses, employing the IVW method, are graphically depicted in **Figure 2**, with the comprehensive results articulated in **Supplementary Table 21**.

### Two-sample MR analysis

**Figure 2** and **Supplementary Table 21** delineated the genetic correlations between AIDs and POI. The MR analyses revealed that genetically determined CeD, vitiligo, SLE, and SIgAD exhibited significant causal relationships with POI. Conversely, no discernible causal links were identified between genetically determined T1DM, CD, PBC, UC, HT, GD, hypothyroidism, AD, PMR, MG, RA, MS, SS, SSc, psoriasis, and ITP with POI.

Specifically, the causal relationship between CeD and POI was explored further. The IVW method revealed a significant positive association, where genetically-inferred predisposition to CeD was linked to an increased likelihood of POI (odds ratio [OR] = 1.124, 95% confidence interval [CI] 1.033-1.224, P = 0.007). This causal effect was corroborated by the weighted median and MR-Egger approaches, which yielded consistent results (**Supplementary Table 21**). Cochran’s Q-test, MR-Egger intercept, and MR-PRESSO analyses, did not indicate the presence of substantial heterogeneity or horizontal pleiotropy (**Supplementary Figure 1**; **Supplementary Table 21**). The leave-one-out sensitivity analysis further confirmed the robustness of the observed causal relationship between genetically-predicted CeD and POI (**Supplementary Figure 2**).

An increased genetic predisposition to vitiligo was associated with a heightened risk of POI, with each 1-unit increase in the log-transformed odds of vitiligo corresponding to an OR of 1.092 (95% CI 1.003-1.188; P = 0.042) for POI. Assessments of horizontal pleiotropy, including MR-Egger intercept and MR-PRESSO, did not reveal any significant biases (**Figure 2**; **Supplementary Table 21**).

Genetically-predicted SLE demonstrated a positive correlation with POI risk using the IVW method (OR = 1.122, 95% CI 1.030-1.223, P = 0.008). Consistent effect directions were observed with the MR-Egger and weighted median approaches, despite some evidence of potential horizontal pleiotropy (MR-PRESSO Global test = 0.044) and heterogeneity (Cochran’s Q test = 0.029). Further investigation identified two outliers (rs150180633 and rs35000415), and after excluding these, the association remained significant (OR = 1.156, 95% CI 1.066-1.254, P < 0.001). Leave-one-out analyses did not reveal any other outliers among the instrumental variables (**Supplementary Figure 3**).

For SIgAD, the MR analysis that was associated with a decreased risk of POI (OR = 0.866, 95% CI: 0.776-0.967, P = 0.011) (**Figure 2**). The result was consistently replicated using the weighted median and MR-Egger approaches, as shown in **Supplementary Table 21**. The causal association between SIgAD and POI demonstrated resilience to biases from horizontal pleiotropic effects (MR-PRESSO Global test = 0.677), and the leave-one-out analysis further validated the reliability of these results (**Supplementary Figure 4**).

Additional methodological replications using expanded GWAS data corroborated the significant causal relationships between genetically-predicted CeD (IVW method: OR = 1.096, 95%CI 1.017-1.182; P = 0.016) (51), SLE (IVW method: OR = 1.220, 95% CI 1.025-1.452, P = 0.025) (27), and vitiligo (IVW method: OR=1.560, 95% CI 1.155-2.107, P = 0.004) (52) with POI (**Supplementary Table 22**). The lack of significant heterogeneity observed in the Cochran’s Q-test and the absence of evidence for horizontal pleiotropy based on the MR-Egger intercept test suggested consistency across the instrumental variables. Furthermore, the leave-one-out sensitivity analysis validated the stability of the results (**Supplementary Figures 5**-**7**).

**Figure 3:**
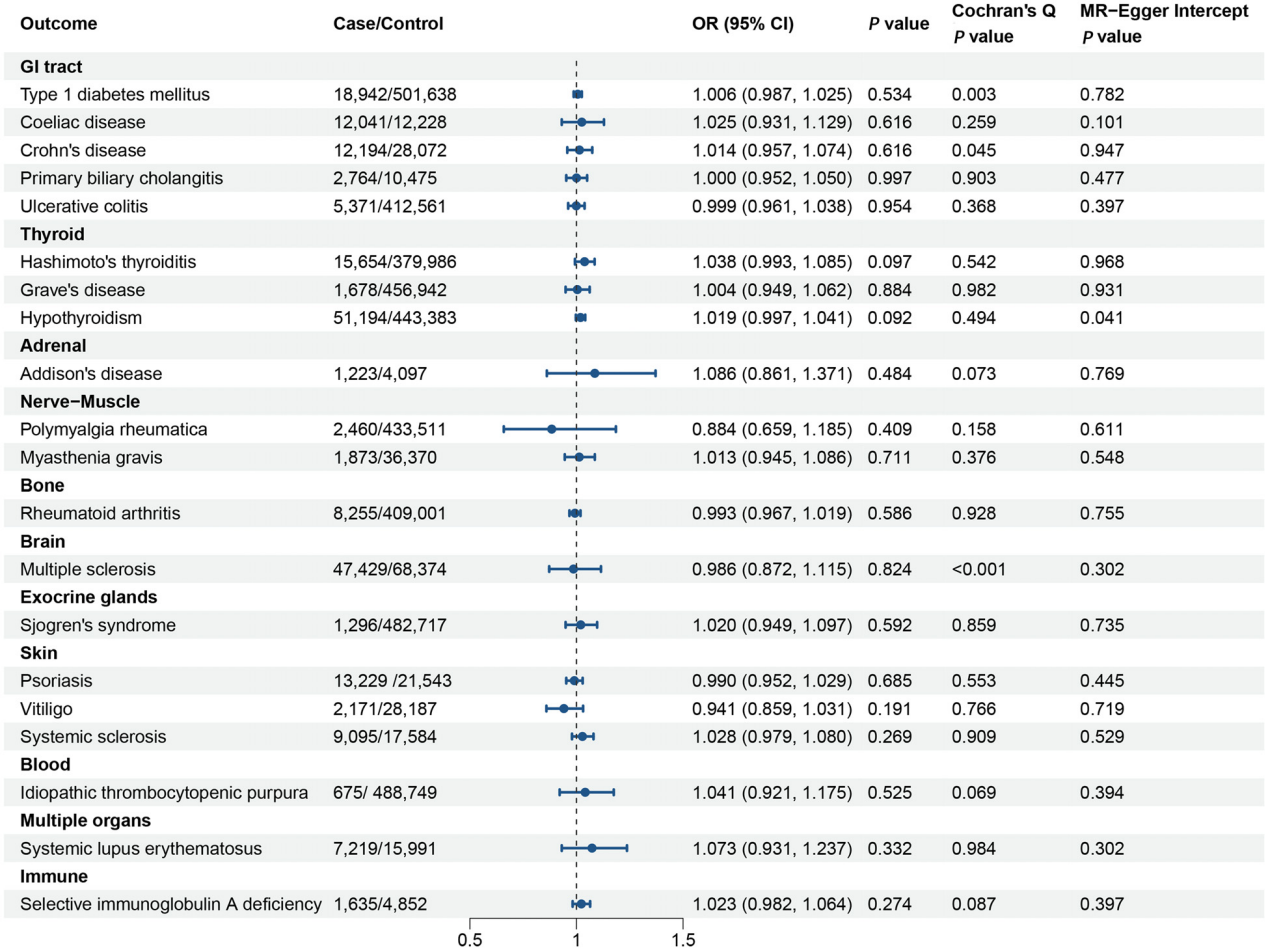
Forest plot illustrating the associations of the genetically predicted primary ovarian insufficiency with the risk of autoimmune diseases. The causal effect of primary ovarian insufficiency on different autoimmune diseases was expressed as OR per unit. Error bars represent the 95% CIs of the estimates. GI, gastrointestinal; OR, odds ratio; CI, confidence interval.

The MR analyses in the reverse direction did not reveal any notable causal effect of genetic predisposition to POI on the susceptibility to AIDs (**Figure 3**). This was observed across various autoimmune diseases, additional details for MR Egger and weighted median methods are provided in the **Supplementary Table 23**.

## Discussion

The present study’s findings from the bidirectional two-sample MR investigation provide novel insights into the causal interplay between a spectrum of autoimmune disorders and primary ovarian insufficiency. By leveraging the robust design of MR, which circumvents the limitations of traditional observational studies, we have delineated a genetic association between certain autoimmune diseases and the susceptibility to POI. Our results revealed a significant genetic predisposition in individuals with coeliac disease, vitiligo, systemic lupus erythematosus, and suggestive evidence of positive effect of Addison’s disease towards POI, indicating that these conditions may predispose to POI at a genetic level. Conversely, selective IgA deficiency was correlated with a decreased incidence of POI, suggesting the presence of a potentially protective genetic factor.

SLE is an autoimmune disease that affects the majority of women in their reproductive years, characterized by an immune system that mistakenly attacks the body’s own tissues, including the ovaries. The relationship between SLE and POI is multifaceted and can involve several mechanisms. The chronic inflammation associated with SLE can lead to tissue damage in the ovaries, potentially resulting in POI. Inflammatory cytokines and immune cells can infiltrate the ovarian tissue and disrupt normal follicular development and function. Certain medications used to treat SLE, particularly alkylating agents like cyclophosphamide (CYC), are known to be gonadotoxic. These drugs can cause a reduction in ovarian reserve and lead to POI. Furthermore, women with SLE frequently possess a variety of autoantibodies that can, in some cases, cross-react with ovarian antigens, potentially triggering an autoimmune assault on the ovaries and resulting in POI. Moreover, there may be genetic and hormonal factors that predispose individuals with SLE to develop POI, certain gene polymorphisms associated with both SLE and POI may contribute to the co-occurrence of these conditions. Additionally, the systemic nature of SLE can lead to a variety of complications that may indirectly affect ovarian function, such as cardiovascular disease, which can influence blood flow to the ovaries. A study by Akawatcharangura et al. reported a heightened incidence of POI within the SLE patient cohort in comparison to that observed in the general population (18). The research reported that 12% of SLE patients treated with immunosuppressive agents developed POI, and a cumulative dose of CYC exceeding 10 grams was the only independent risk factor for POI. The study suggests that systematic evaluation and early recognition of POI should be promoted in the care of SLE patients to prevent these events. Another study also pointed out that in the absence of CYC treatment, the prevalence of POF in SLE patients is consistent with that of the general population (53). This indicates that most SLE patients are unlikely to have autoimmune processes in the ovaries. Consistent with these researches, our study revealed that SLE as a risk factor for POI among women of reproductive age. These findings underscore the importance of monitoring ovarian function in SLE patients, especially those who are undergoing treatments with potential gonadotoxic medications that may affect ovarian reserve (54). For women with SLE, particularly those planning for future fertility, understanding and assessing the risk of POI is crucial.

Vitiligo is a chronic skin condition characterized by the appearance of white patches due to the loss of melanocytes, the pigment-producing cells in the skin. It affects both genders and involves complex etiological factors, including genetics, autoimmune responses, oxidative stress, and melanocyte destruction. Both innate and adaptive immune systems play a significant role in the pathogenesis of vitiligo. Melanocytes, the pigment-producing cells in the skin, are destroyed through mechanisms involving molecules released from these cells and possibly keratinocytes, which trigger an immune response and target various organs, including the ovaries, leading to a decrease in ovarian reserves. Ünal et al. investigated the relationship between vitiligo and its potential effects on ovarian reserves in women (55). The study found a negative correlation between the duration of vitiligo and AMH, prolactin, and ovarian volume, indicating that longer exposure to the disease may be associated with reduced ovarian reserve. A positive correlation was also observed between disease duration and LH and POI rates. We found a positive causal association between vitiligo and POI in our MR study (OR=1.092, 95% CI 1.003-1.188, P=0.042). The result is consistent with forementioned case-control study (55). Given the potential autoimmune link between vitiligo and POI, it is recommended that once vitiligo is diagnosed, the ovarian reserve should be evaluated and monitored routinely in all reproductive-aged women. Early detection and appropriate management of these conditions can help improve the quality of life and reproductive health for affected individuals.

Celiac disease, characterized by an immune-mediated intolerance to gluten, can lead to systemic effects that extend beyond the gastrointestinal tract, including potential endocrinopathies that may affect ovarian function (56). The potential impact of celiac disease on ovarian reserve may be mediated through several mechanisms. Malnutrition, particularly deficiencies in vitamins and minerals such as iron, zinc, and vitamin D, which are common in celiac disease, can disrupt the hypothalamic-pituitary-ovarian axis, leading to hormonal imbalances and potentially affecting follicular development and ovulation. Additionally, chronic inflammation and the immune response associated with celiac disease may have direct effects on ovarian tissue. However, research on this topic has yielded mixed results. Cakmak et al. found that AMH level and ovarian reserve was decreased and their decline is correlated with the duration of the celiac disease in patients of reproductive age (57), while Comba et al. reported no significant impact in adolescent girls (58). It is possible that the influence of celiac disease on ovarian reserve may vary depending on factors including the duration and severity of the disease, the adequacy of dietary management, and individual genetic predispositions. In our study, we observed that celiac disease increased the risk of POI (OR=1.124, 95% CI: 1.033 to 1.224, P=0.007), and this result was similar upon validation in a larger dataset (OR=1.096, 95% CI: 1.017 to 1.182, P=0.015). In light of these considerations, it is prudent for clinicians to be vigilant in assessing risk of POI in women with celiac disease of reproductive age, particularly those with a history of poor disease control or nutritional deficiencies. Early detection and appropriate management of celiac disease, including the implementation of a gluten-free diet and supplementation to correct nutritional deficiencies, may be crucial in preserving ovarian reserve and optimizing fertility outcomes.

Selective IgA Deficiency (SIgAD) is a primary immunodeficiency disorder characterized by the near absence or significantly reduced levels of immunoglobulin A in the blood. It is the most common form of primary immunodeficiency. While the majority of individuals with SIgAD exhibit an asymptomatic course, a subset of patients may experience a spectrum of clinical sequelae, including respiratory infections, allergic reactions, gastrointestinal tract pathologies, neoplastic conditions, and autoimmune disorders. Magen et al. reported that three patients were diagnosed with SIgAD and presented with amenorrhea and desired fertility and suggested that POI may be one of the concomitant autoimmune diseases in SIgAD patients (59). Our MR study indicated a negative association between SIgAD and POI. These studies highlighted the potential link between SIgAD and POI, emphasizing the importance of considering autoimmune etiologies in the differential diagnosis of POI, especially in patients with a history of primary immunodeficiencies.

Addison’s disease is characterized by the insufficient production of glucocorticoids by the adrenal cortex, leading to a range of symptoms including fatigue, hypotension, and hyperpigmentation. The coexistence of AD and POI is more frequent than would be expected by chance, suggesting a shared pathophysiological mechanism. Both conditions are part of the spectrum of autoimmune endocrinopathies and are associated with the presence of specific autoantibodies. For instance, antibodies against 21-hydroxylase, an enzyme crucial for steroid synthesis in the adrenal and ovarian tissues, are commonly found in patients with AD and can also be present in those with POI (60). Recent research has further highlighted the association between AD and POI. Vogt et al. found that women with AD have a higher prevalence of POI compared to the general population (61). This study underscores the importance of screening women with AD for POI and vice versa, given the potential impact on fertility and long-term health. Another study offered insights into the disease progression of autoimmune POI in individuals with AD, delineating the transition from normal ovarian function to manifest ovarian dysfunction (62). This research emphasizes the dynamic nature of autoimmune endocrinopathies and the need for longitudinal follow-up to monitor changes in ovarian function. In our investigation, we observed that the suggestive evidence of adverse effect of AD on the susceptibility to POI were generally in line with existing observational research; however, this association did not remain significant following the implementation of more rigorous selection criteria for instrumental variables. In conclusion, the relationship between AD and POI is complex and multifaceted, with autoimmune mechanisms playing a pivotal role in the pathogenesis of both conditions. The increased prevalence of POI in women with AD highlights the importance of a comprehensive endocrine evaluation and appropriate management strategies to preserve reproductive health and overall well-being.

Among autoimmune-related etiologies, thyroid dysfunctions, specifically Hashimoto’s thyroiditis, hypothyroidism, and Graves’ disease, exhibit the correlation with POI (16, 63), but the findings have differed considerably across studies, and conclusive evidence has remained elusive (64, 65). Recently, Hsieh et al. analyzed 21,325 subjects with autoimmune thyroid diseases (Hashimoto’s and Grave’s disease) and found that they are associated with higher risk of POI (66). However, Polyzos et al. conducted a study recruiting 5000 women and reported that neither thyroid autoimmunity (TAI) nor hypothyroidism was associated with poor ovarian reserve (67). In our MR study, we observed that hypothyroidism, Hashimoto’s thyroiditis, and Graves’ disease increased the risk of POI but the results were not statistically significant.

To the best of our knowledge, this investigation marks the first application of MR to elucidate the causal impact of autoimmune diseases on the POI, utilizing the most contemporary and extensive GWAS data available, ensuring a broad representation of the European population. The MR approach was employed to circumvent potential confounding biases, thereby facilitating the derivation of robust causal inferences. The credibility of our findings is further bolstered by a series of sensitivity analyses. These analyses meticulously excluded SNPs that are correlated with cigarette smoking from the set of instrumental variables (68). This exclusionary approach ensures a more stringent assessment of the MR results, mitigating the potential influence of these SNPs on the observed associations. The MR analyses yielded consistent findings across various methodological approaches, including the application of simple median, weighted median, and MR-Egger regression models. The robustness of the associations was further substantiated by leave-one-out sensitivity analyses, which demonstrated stable relationships upon sequential exclusion of individual SNPs, although the specific data for these analyses are not presented. Despite the MR-Egger regression not revealing evidence of either beneficial or detrimental pleiotropy, the possibility of balanced pleiotropy remains an unresolved concern.

Nevertheless, the study acknowledges several limitations that warrant emphasis. Initially, the reliance on genetic data predominantly from individuals of European descent in GWAS may restrict the applicability of the research outcomes across heterogeneous populations. Furthermore, despite rigorous attempts to address pleiotropy, complete eradication of all forms of pleiotropic effects in MR studies remains unattainable. Consequently, unexplored pathways and confounding variables may persist, potentially introducing bias into the outcomes. Lastly, a notable constraint of this study is the inability to stratify the analysis according to the severity of autoimmune disorders and other critical factors, such as age, which could offer a more nuanced comprehension of the examined associations. Fourthly, sex-specific aggregated data from GWAS pertaining to autoimmune diseases were inaccessible. However, a population-based cohort study of 22 million individuals in the UK reported that most autoimmune disorders were more common in women than in men (69). Thyroid disorders, Sjögren’s syndrome, systemic lupus erythematosus, and systemic sclerosis had the highest incidence rate ratios in women compared with men (69). To rule out reversed causation, these analyses should be rerun in women and men separately.

In conclusion, the comprehensive MR analysis conducted in this study indicated that SLE, CeD, vitiligo, and AD caused an increased risk of POI, SIgAD was associated with a decreased risk of POI. These insights carry profound clinical implications, particularly emphasizing the early intervention for women with AIDs/POI who wish to preserve their reproductive potential or plan for future pregnancies.

## Data Availability

The original contributions presented in the study are included in the article/**Supplementary Material**. Further inquiries can be directed to the corresponding authors.
