# Supplementary figures and images for: Primary ovarian insufficiency consequence of autoimmune diseases: a bidirectional two-sample Mendelian randomization study
Source: Front Endocrinol (Lausanne). 2024 Dec 9;15:1417896. doi: 10.3389/fendo.2024.1417896 (PMC11663653; doi:10.3389/fendo.2024.1417896)

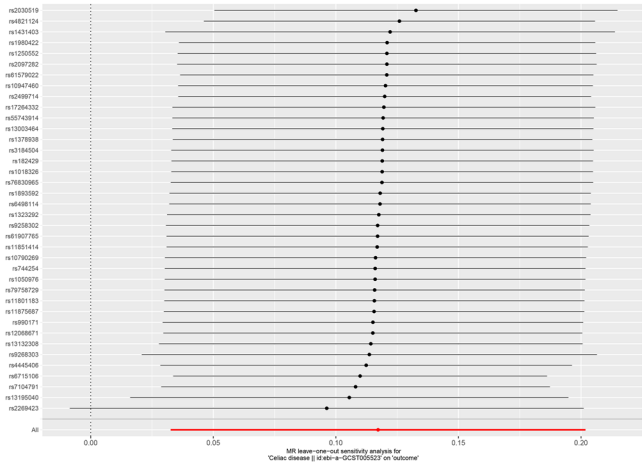

Supplement: Supplementary file 1 [file DataSheet1.pdf]

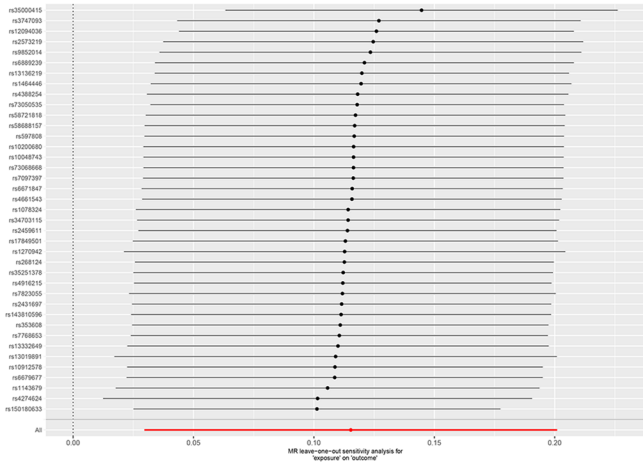

Supplement: Supplementary file 2 [file DataSheet2.pdf]

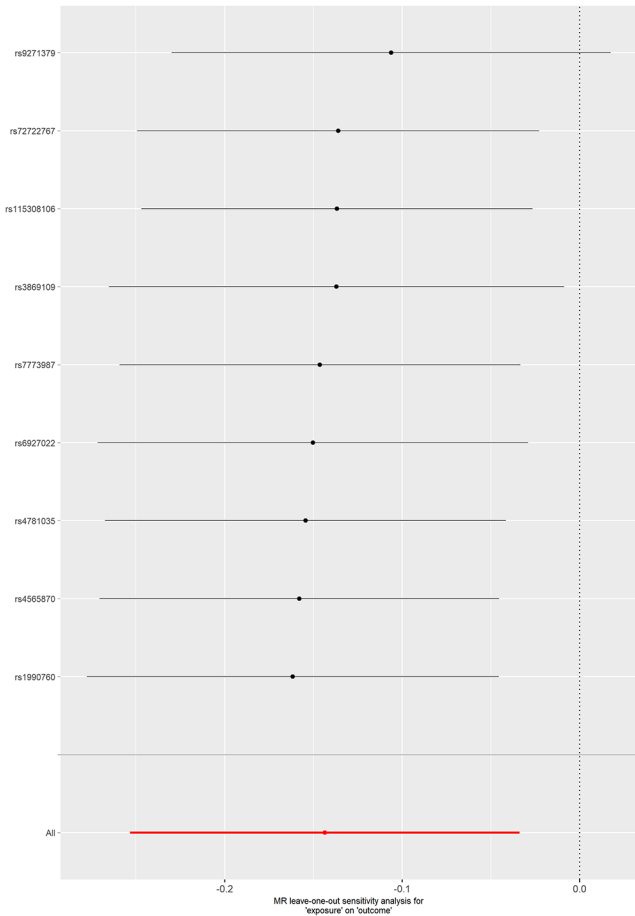

Supplement: Supplementary file 3 [file DataSheet3.pdf]

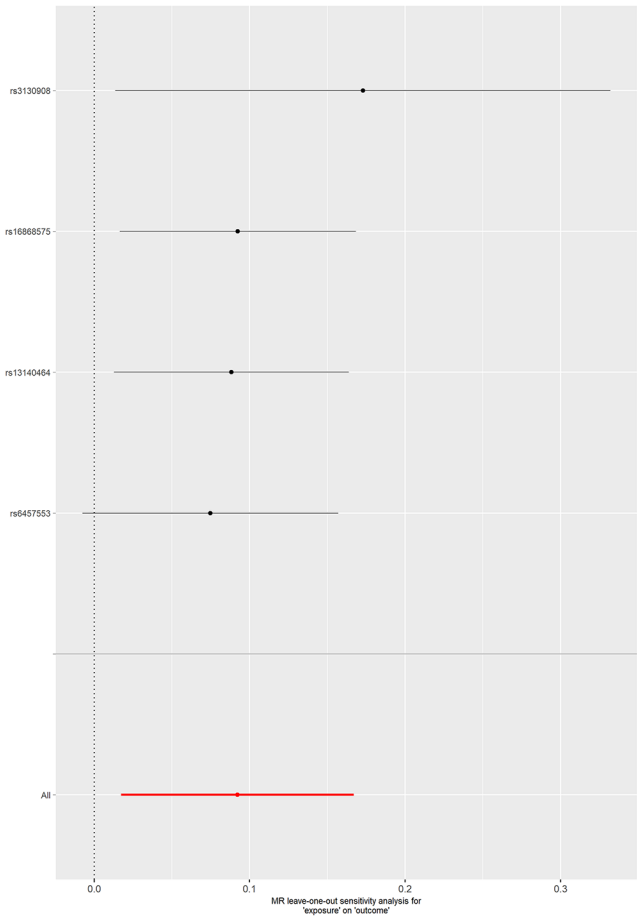

Supplement: Supplementary file 4 [file DataSheet4.pdf]

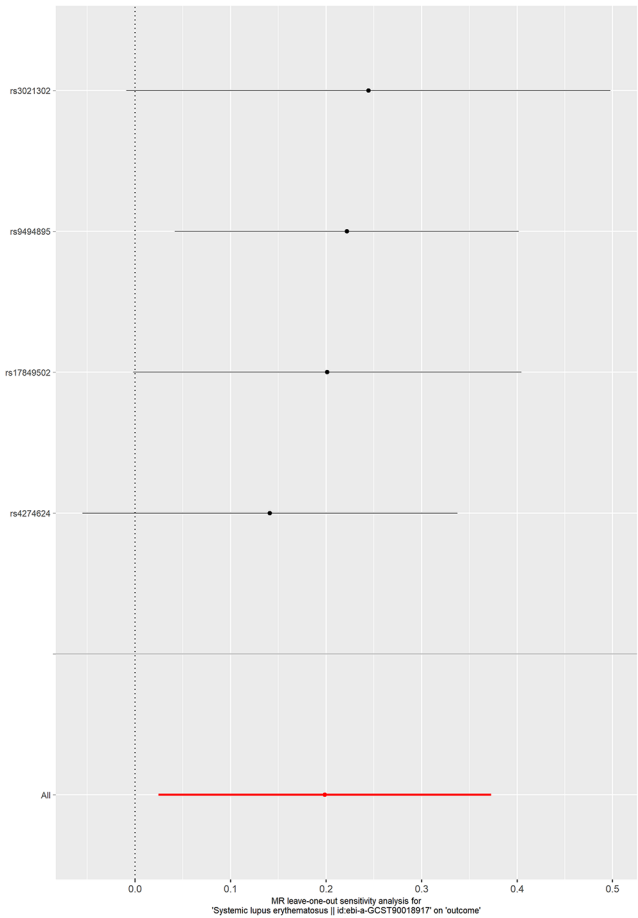

Supplement: Supplementary file 5 [file DataSheet5.pdf]

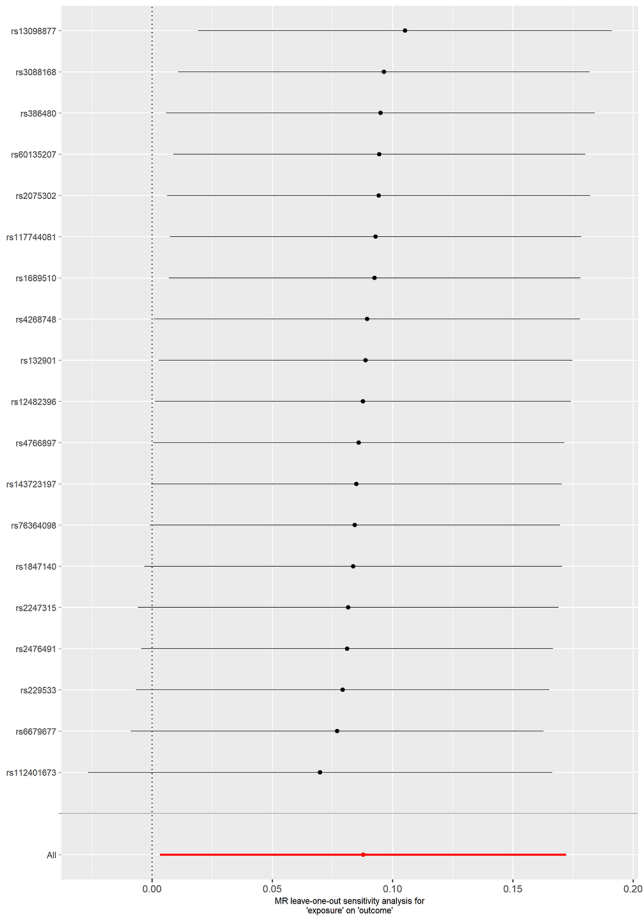

Supplement: Supplementary file 6 [file DataSheet6.pdf]
