# Supplementary material for: Primary ovarian insufficiency consequence of autoimmune diseases: a bidirectional two-sample Mendelian randomization study
Source: Front Endocrinol (Lausanne). 2024 Dec 9;15:1417896. doi: 10.3389/fendo.2024.1417896 (PMC11663653; doi:10.3389/fendo.2024.1417896)
Supplement: Supplementary file 9 [file DataSheet9.docx]

**Figure legends**

**Supplementary Figure 1.** Funnel plot showed the causal relationship between CeD and POI from three different methods.

**Supplementary Figure 2.** Leave-one-out plot of the sensitivity analysis on the causal association of genetically predicted CeD with POI.

**Supplementary Figure 3.** Leave-one-out plot of the sensitivity analysis on the causal association of genetically predicted SLE with POI.

**Supplementary Figure 4.** Leave-one-out plot of the sensitivity analysis on the causal association of genetically predicted SIgAD with POI.

**Supplementary Figure 5.** Leave-one-out plot of the sensitivity analysis on the causal association of genetically predicted CeD with POI in the replication analysis.

**Supplementary Figure 6.** Leave-one-out plot of the sensitivity analysis on the causal association of genetically predicted SLE with POI in the replication analysis.

**Supplementary Figure 7.** Leave-one-out plot of the sensitivity analysis on the causal association of genetically predicted vitiligo with POI in the replication analysis.
